# Plants’ Physio-Biochemical and Phyto-Hormonal Responses to Alleviate the Adverse Effects of Drought Stress: A Comprehensive Review

**DOI:** 10.3390/plants11131620

**Published:** 2022-06-21

**Authors:** Abdul Wahab, Gholamreza Abdi, Muhammad Hamzah Saleem, Baber Ali, Saqib Ullah, Wadood Shah, Sahar Mumtaz, Ghulam Yasin, Crina Carmen Muresan, Romina Alina Marc

**Affiliations:** 1 Shanghai Center for Plant Stress Biology, CAS Center for Excellence in Molecular Plant Sciences, Chinese Academy of Sciences, Shanghai 200032, China; wahabcrop_science@mails.ucas.ac.cn; 2 Department of Biotechnology, Persian Gulf Research Institute, Persian Gulf University, Bushehr 75169, Iran; abdi@pgu.ac.ir; 3 College of Plant Science and Technology, Huazhong Agricultural University, Wuhan 430070, China; 4 Department of Plant Sciences, Quaid-i-Azam University, Islamabad 45320, Pakistan; baberali@bs.qau.edu.pk; 5 Department of Botany, Islamia College, Peshawar 25120, Pakistan; saqibullahstd@icp.edu.pk; 6 Department of Botany, University of Peshawar, Peshawar 25120, Pakistan; wadood0301@gmail.com; 7 Department of Botany, Division of Science and Technology, University of Education, Lahore 54770, Pakistan; sahar_botany@yahoo.com; 8 Department of Botany, Bahauddin Zakariya University, Multan 60800, Pakistan; yasingmn_bzu@yahoo.com; 9 Food Engineering Department, Faculty of Food Science and Technology, University of Agricultural Science and Veterinary Medicine Cluj-Napoca, 3-5 Calea Mănăştur Street, 400372 Cluj-Napoca, Romania; crina.muresan@usamvcluj.ro

**Keywords:** drought stress, abiotic stress, osmolytes, antioxidant enzymes, phytohormones, photosynthesis

## Abstract

Water, a necessary component of cell protoplasm, plays an essential role in supporting life on Earth; nevertheless, extreme changes in climatic conditions limit water availability, causing numerous issues, such as the current water-scarce regimes in many regions of the biome. This review aims to collect data from various published studies in the literature to understand and critically analyze plants’ morphological, growth, yield, and physio-biochemical responses to drought stress and their potential to modulate and nullify the damaging effects of drought stress via activating natural physiological and biochemical mechanisms. In addition, the review described current breakthroughs in understanding how plant hormones influence drought stress responses and phytohormonal interaction through signaling under water stress regimes. The information for this review was systematically gathered from different global search engines and the scientific literature databases Science Direct, including Google Scholar, Web of Science, related studies, published books, and articles. Drought stress is a significant obstacle to meeting food demand for the world’s constantly growing population. Plants cope with stress regimes through changes to cellular osmotic potential, water potential, and activation of natural defense systems in the form of antioxidant enzymes and accumulation of osmolytes including proteins, proline, glycine betaine, phenolic compounds, and soluble sugars. Phytohormones modulate developmental processes and signaling networks, which aid in acclimating plants to biotic and abiotic challenges and, consequently, their survival. Significant progress has been made for jasmonates, salicylic acid, and ethylene in identifying important components and understanding their roles in plant responses to abiotic stress. Other plant hormones, such as abscisic acid, auxin, gibberellic acid, brassinosteroids, and peptide hormones, have been linked to plant defense signaling pathways in various ways.

## 1. Introduction

### Drought Stress

Changing climatic regimes are posing a threat to life on Earth because meeting the rising food demand and achieving sustainable agriculture for a growing population is becoming an uphill task in the present scenario of changing climatic conditions [1], which include droughts, heavy floods, earthquakes, and temperature variations [2,3]. Drought stress interrupts many physio-biochemical processes, hindering plant growth and development [4,5]. Plants can frequently withstand limited water conditions but at the cost of substantial loss in total biomass and productivity. Drought affects around half of the world’s semi-arid and arid areas. Photosynthesis, growth, and other critical physiological and biochemical activities are interrupted under drought stress conditions [6,7]. Previous studies [8,9,10] found that drought stress causes oxidative stress, damaging biological membranes and macromolecules (DNA, proteins, lipids, and photosynthetic pigments). Plants engage their natural defense systems in response to oxidative stress and create osmolytes [11], such as soluble proteins, proline, soluble sugars, and glycine betaine [12].

**Figure 1:**
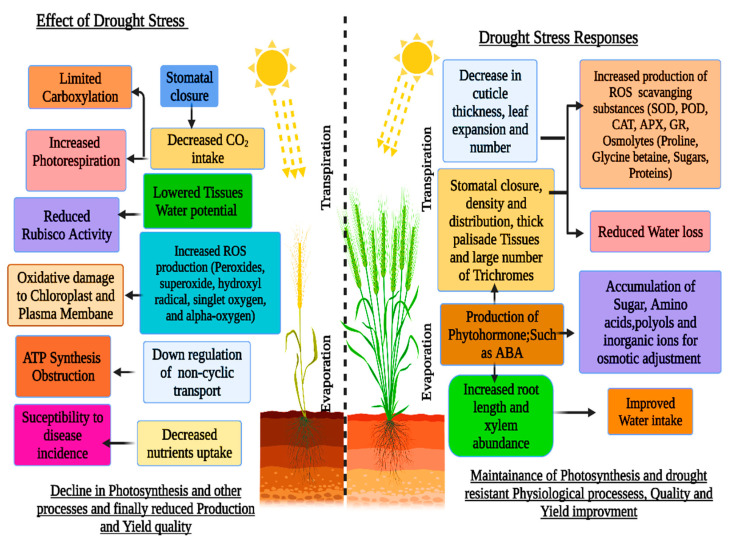
Effects of drought stress on sensitive and tolerant wheat (*Triticum aestivum* L.) crops.

Osmolytes, also known as osmoprotectants, are found mainly in the cytoplasm and prevent cellular deterioration by maintaining the cell’s osmoregulation. Because osmolytes are non-toxic and highly soluble, they do not interfere with other physiological and biochemical processes [13,14]. Plants generate antioxidant substances such as flavonoids, carotenoids, vitamins, and antioxidative enzymes such as glutathione reductase (GTX), superoxide dismutase (SOD), catalase (CAT), peroxidase (POD), and ascorbate peroxidase (APX) in response to abiotic stress [15,16]. Water deprivation causes reduced turgor pressure and oxidative damage from reactive oxygen species (ROS), including superoxide and hydroxyl radicals, nitric oxide and singlet oxygen, causing alterations in leaf gas exchange rates [17] (Figure 1). Natural drought-resistance mechanisms in plants have been well developed, including morphological, physiological, and biochemical adaptations, such as drought-resistant epigenetic plasticity and gene activation [18]. Drought resistance and transformation in food legumes and crop plants are maintained through morphological, physiological, and biochemical changes. These characteristics may assist crops in adapting to harsh environmental conditions. Imbalances in nutrition are caused by drought stress, causing significant ecological constraints on agricultural output worldwide [19].

**Figure 2:**
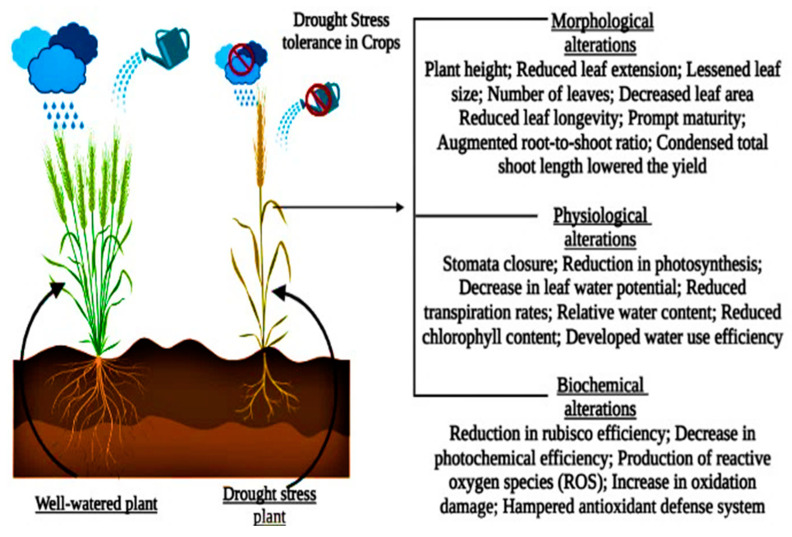
Drought stress impacts plants’ morphological, physiological, and biochemical processes.

Drought stress is the most challenging issue to agricultural productivity and has a pronounced negative effect on plant growth, development, and productivity. Making it difficult to maintain a sustainable agricultural system worldwide [19,20]. Drought-induced changes in wheat characteristics were investigated, and their impact on agronomic attributes and yield were studied. Spikelet fertility and grain filling were affected negatively by drought stress [21]. Maize (*Zea mays* L.) and wheat (*Triticum aestivum* L.) crops water limitations lead to reduced crop yields and quality]. Water stressed conditions reduce agricultural output and put food production at risk [22,23]. Reduction in agricultural productivity leads to shrinking revenue for local farmers. The loss of production substantially impacts farmers’ livelihoods and economies [24]. Wheat (*Triticum aestivum* L.) crop is a major cereal crop and a common food source worldwide. Wheat (*Triticum aestivum* L.) crop with improved drought tolerance is essential for long-term food production and global food security [25]. Many critical genes and transcription regulators controlling morpho-physiological and biochemical features have been discovered due to recent developments in drought tolerance research [26] (Figure 2).

## 2. Drought-Induced Changes in Plant Morphology

Drought stress adversely affects morphological aspects of plants, such as early germination, plant height, relative root length, root diameter, the total biomass of leaves and roots, number of leaves/plants, and branch number/plant [27,28].

### 2.1. Early Seed Germination and Flowering

Water is essential for seed germination; however, while other conditions may be ideal, drought stress inhibits the imbibition of seeds and, consequently, hinders germination [29]. Similarly, it reduces seedling vigour and impacts germination by lowering water intake [30]. In the early stages of crop development, drought stress manifests through reduced seed germination resulting in poor stand establishment [31]. Poor seedling germination was observed under exposure to drought stress in two crops: rice (*Oryza sativa* L.) and pea (*Pisum sativum* L.) [30,32]. Low water content in the soil combined with other environmental factors can alter germination success. Drought stress considerably influences *Zea mays* L. seedling germination [33,34]. Some field crops are particularly vulnerable to cold and dryness, especially during germination and seedling development (early phases). Every seed has optimal soil moisture levels and temperature for germination [35].

### 2.2. Plant Morphological Characteristics of Leaves under Drought Stress

Drought stress substantially influences the internal plant components that increase plant height [35]. Plant height loss might be related to decreased cell growth, a high rate of leaf abscission under dryness, and poor mitosis [36,37]. Water stressed conditions considerably reduced the number of leaves in *Zea mays* L. [34,38]. The study by [33] showed that sweet basil (*Ocimum basilicum*) leaves are significantly more critical than shoot and roots because leaves are responsible for photosynthesis and contain photosynthetic pigments. Drought regimes reduce leaf area and plant total biomass [39]; by limiting leaf growth and affecting the photosynthetic process. Previous research studies reported that leaf area was significantly decreased under drought stress conditions in many crops, including *Triticum aestivum* L. and *Oryza sativa* L. [39,40]. Loss of water from the upper epidermis of the leaf results in diminishing leaf pressure potential, which causes the leaf to roll. Reduced leaf temperature, increased interception of the incident light, and increased transpiration rate benefit this phenomenon. Under drought stress regimes, leaf area and leaf rolling were dramatically enhanced in maize (*Zea mays* L.) crop leaves [41].

### 2.3. Plants Shoot Morphology and Architecture under Drought Stress

Drought stress has a negative impact on shoot length and fresh weight. In *Phaseolus vulgaris* L., however, there was a considerable drop in the dry weight of the shoot [42]. Conversely, the shoot length in maize (*Zea mays* L.) crop was discovered, which needs to recover by supplying adequate water and nutrients for survival and defeating drought stress conditions [43]. Similarly, it was observed that the seedling length of maize (*Zea mays* L.) was dramatically reduced under drought stress. The water shortage tremendously affects maize crops’ dry weight after drying in shades [44]. The results showed drought stress considerably affects maize (*Zea mays* L.) crops’ fresh weight compared to control. In such situations, the plant needs a well-developed root system attaching themselves and collecting water and nutrients from their environment [45].

### 2.4. Plant Root Morphology and Architecture under Drought Stress

Drought stress alters agricultural plants’ root architecture and morphology. During abiotic stress conditions, many plants’ root biomass increases as the roots’ length become more prolonged, and more water and minerals are absorbed from the soil [18,46]. Furthermore, polyethylene glycol-induced drought stress decreased hypocotyl length and fresh and dry weight roots in maize (*Zea mays* L.) while increasing root length [47]. Sometimes, moderate drought has no pronounced negative impacts on root development [48]. Root development in maize, for example, was unaffected by water stress [49]. Previous research found that drought stress boosted root development in two plants, *Catharanthus roseus* L. and *Helianthus anuus* L. [49]. Drought stress affects crops, but the most relevant characteristic is increased legumes, shoot, and root-shoot ratios in different plant species [16]. Root architecture plays a crucial role in plant growth and development. When plants are subjected to water-stressed conditions, their roots elongate into the ground, reaching deeper and absorbing enough water and minerals to survive [43].

### 2.5. Yield

In some plant species, yields may be reduced depending on the period and intensity of the limited water condition; nevertheless, the lack after anthesis is deleterious to crop yields regardless of the severity and time of the deficit. Drought stress lowers yields in several ways [50]. In barley (*Hordeum vulgare* L.) and wheat (*Triticum aestivum* L.) crops, drought stress reduced the number of spikes, tillers, and grains per plant, as well as the grain weight [51,52]. Reduced millet (*Pennisetum glaucum* L.) production by drought stress caused silking to be delayed and the anthesis-to-silking gap to be lengthened [53]. Drought stress harmed soybean seed production and influenced the physiology and yield of crop germplasms [50]. This trait was substantially related to grain production, namely the number of ears and kernels per plant [54]. The association was investigated between grain yield, grasslands, and harvest index [55].

**Figure 3:**
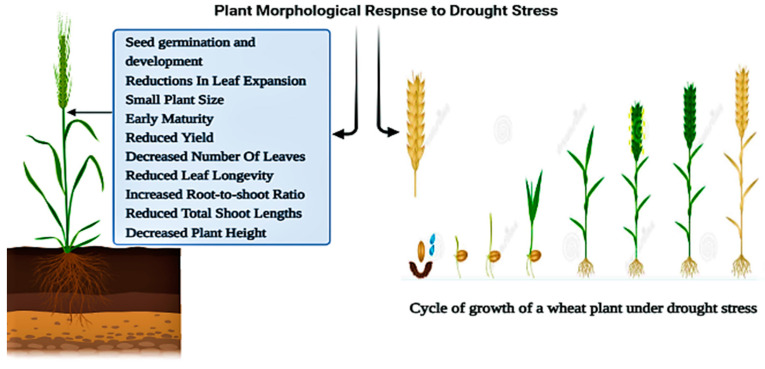
Impact of drought stress on morphological aspects: Cycle of growth of a *Triticum aestivum* L. plant.

Here we are discussing that drought stress dramatically impacts plants’ morphological characteristics of wheat corps (*Triticum aestivum* L.). Drought stress significantly affects the early stages of seed germinations [56]. In leaf morphology, drought stress also plays a critical role, such as; a reduction in leaf expansions and leaf rolling [57]. Productions of Yield quality and yield losses in wheat crop (*Triticum aestivum* L.) species have been linked to a limited water conditions, with the severity and duration being the focus factors in this association, as shown in Figure 3 [58].

## 3. Physiological and Biochemical Responses under Drought Stress

**Figure 4:**
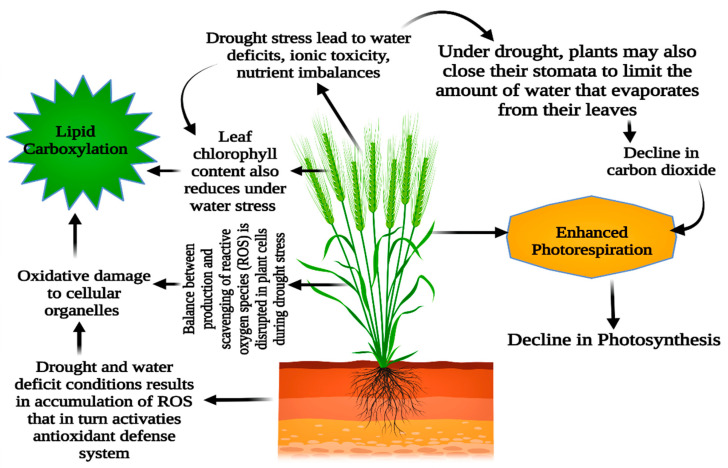
Drought stress’s effects on *Triticum aestivum* L. plant morpho-physiological and metabolic processes.

Drought causes water shortage since there is not enough water in the soil. A water shortage in the soil is not always the cause of the physiological drought [59]. A physiological drought occurs when a plant cannot get enough water; plants react to water stress in various ways [50]. Physiological, biochemical, anatomical, morphological, and long- and short-term developmental and growth-related adaptable techniques might be involved (Figure 4) [60,61,62]. Reducing leaf relative water content, turgor loss, and stomatal closure are the frequent consequences of drought stress in Barley (*Hordeum vulgare* L.) [51]. During drought, leaf wilting and abscission reduce water loss via transpiration [17,63]. When there is a significant water shortage, cell enlargement in higher plants is hampered by the interruption of xylem water flow. When drought stress is minimal to nonexistent, stomatal closure, cell membrane structural damage, and plant metabolic disturbances occur [64,65]. The results suggested by [43] concluded that numerous internal and external conditions govern internal plant water interactions in *Zea mays* L., such as the stomatal resistance, RWC, rate of transpiration, leaf temperature of wheat crop (*Triticum aestivum* L.), leaf water potential, and the canopy temperature just above the plant [21,66,67].

### 3.1. Leaf Relative Water Content (RWC)

Leaf RWC is a crucial controller of physiological processes in plants. RWC reduction is the first symptom of the drought stress response [68]. The relative water content of leaves strongly correlates simultaneously with leaf tissue growth rate and rate of transpiration [69]. Lower RWC reduces leaf water potential, causing stomata to contract. Transpiration is the primary mechanism governing leaf temperature; increasing stomatal resistance minimizes the transpiration rate in rice leaves due to ABA content and increases leaf temperature [63]. In a *Triticum aestivum* L., the leaf’s relative water content increases throughout development and decreases as dry matter accumulates as the leaf ages [70]. Water-stressed wheat and rice plants contained less water content as compared to those wheat and rice plants that were grown under controlled conditions [71]. A decline in relative water content induced a drop in water content and osmotic potential under stress regimes. In wheat (*Triticum aestivum*), the state of reduced leaf turgor pressure disrupts plant metabolic functions. Under drought conditions, crop development is impeded by a lower soil water potential, and the resulting lowered plant osmotic potential leads to low nutrient absorption [72].

### 3.2. Effect of Drought Stress Conditions on Photosynthesis and Stomatal Aperture

**Figure 5:**
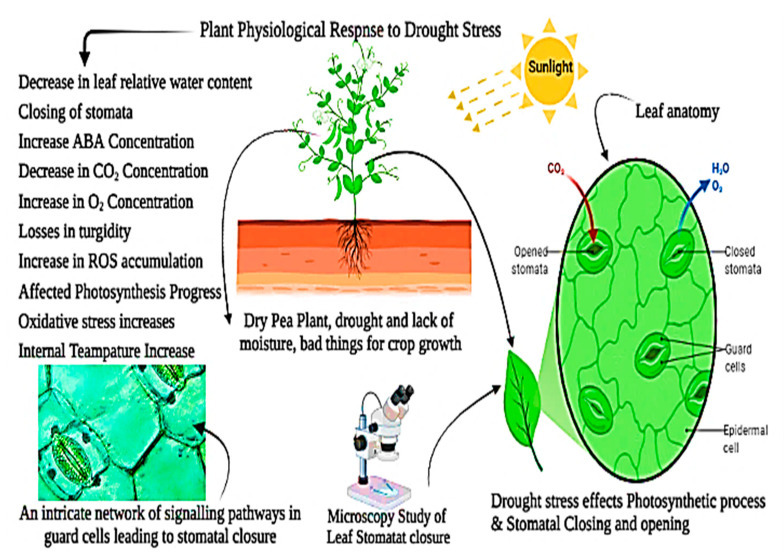
Drought stress and morpho-physiological responses in pea plants; drought stress affects photosynthetic pigments and leaf stomatal openings and closings in pea crops (*Pisum sativum* L.).

In photosynthesis, CO_2_ and H_2_O within the chloroplast of plant cells produce sugars and O_2_ as a by-product in the presence of light. Chlorophyll is an essential component of chloroplasts required for photosynthesis [73,74]. Chlorophyll pigments are essential for photosynthesis, affected by water-stressed conditions during stomatal closure and openings in *Nicotiana tabacum* L. [75]. Plants must capture light and use it during the photosynthesis process. Under drought stress, the chlorophyll concentration is dramatically reduced due to increased oxidative stress, degeneration, or photo-oxidation of chlorophyll pigments [76]. Drought sensitivity in (*Triticum aestivum* L.) was predominantly connected with reductions in stomatal conductance, which decreased the delivery of carbon dioxides to chloroplasts and, consequently, reduced net photosynthesis [77,78]. The results determined that drought stress affected plant growth and development by lowering the rate of photosynthesis [79]. The major factors responsible for slowing photosynthesis might be stomatal closure (reduced stomatal CO_2_ fixation), non-stomatal (decreased photosynthesis activity in mesophyll tissues), or both [80,81]. Water stressed condition is one of the numerous environmental variables that impede photosynthesis. The high sensitivity of connecting photosystems II (PS-II) following limiting tensions induced by external variables motivates drought stress in harming these systems, which are reaction locations. Methods of chlorophyll fluorescence revealed a hazard and suggested that manufacturing operations were not balanced [82,83]. Drought stress causes plants to be adapted accordingly by regulating their stomata movement, adjusting their osmotic balance, and mounting an antioxidatant defense [8,84]. However, a protracted period of high-intensity limited water conditions might slow plant development, alter the morphological structure and biomass distribution pattern in tomato crops (*Solanum lycopersicum* L.), or cause mortality [15,85]. Drought stress significantly influences the photosynthetic system and its pigments, such as chlorophyll a, b, and carotenoids [72,86,87]. Drought stress also impacts complex systems such as photosystems I and II. Drought stress significantly influences plant starch production by affecting the Calvin cycle and enzyme activity (*Ribulose phosphate*) [86]. The first sign of a plant’s drought stress response is closing its stomata. When drought stress becomes more severe during the day, stomata progressively close in sugar beet (*Beta vulgaris* L.) [88]. Stomata are entirely closed in extreme drought stress conditions. Still, full closure varies among plant species depending on their specific tolerances to drought conditions, as shown in pea crops (*Pisum sativum* L.) [30,89] (Figure 5). As a result, plant species tolerance influences the stomatal mechanism, which regulates carbon fixation rates, photosynthesis, and water usage efficiency. When stomata restrict CO_2_ uptake into the leaves, more electrons are available to produce active oxygen species [90]. When physiological processes at the stomata are reduced by environmental conditions that increase transpiration rates, then the pH of the leaf sap is elevated; [91] observed reductions of photosynthesis, ROS production regulations decreased, and stomatal conductance under drought stress could be recovered by following re-watering [92].

### 3.3. Carotenoids

According to [34,93] studies, drought stress has reduced the concentration of carotenoids in higher plants. Carotenes are classified into two types: hydro-carbon carotenes [83], which include lycopene and xanthophylls, and carotene, which differs from the former due to the inclusion of lutein. The enzymatic antioxidant system contains carotenes, tocopherol, ascorbate, and enzymes such as APX, POD, SOD, polyphenol oxidase, glutathione reductase (GR), and CAT was, protecting carotenoids from the damaging of ROS [8,88,94,95,96,97]. The enzymatic antioxidant system, which contains carotenoids, also protects carotenoids from ROS. Beta-carotene, which is involved in the breakdown of triple chlorophyll, prevents singlet oxygen formation, which helps in protecting the plant cells from oxidative stress. In addition, carotene is required to avoid and maintain photochemical reactions [14].

### 3.4. Cell Size, Cell Membrane Stability, and Respiration

Many developmental processes and all aspects of the growth have been adversely affected by droughts, such as cell division, cell expansion, cell differentiation, and genetic, ecological, and physio-morphological approaches [57]. These events, influenced by limited water regimes, govern the amount and quality of plant growth. As a result of the drought, one of the most drought-sensitive physiological processes is cell development as turgor pressure drops [62]. Drought stress is characterized by the limitations of a water path from the xylem to the neighboring elongating cells, which ultimately results in the plant’s death; it may impair cell elongation in higher plants [95]. According to [22], drought stress reduces cell size in winter wheat crops (*Triticum aestivum* L.) verities; and enhances interactions between Protein-protein aggregation and denaturation [96]. It is possible that increasing solute concentrations, particularly in the presence of photosynthetic equipment, will be harmful to enzyme activity, as evidenced by an increase in cytoplasmic viscosity [57]. Drought stress reduces the respiration rate in various plant components, including leaves, shoots, and the whole plant [15,97]. According to research, plants’ respiration rates remain unaltered or even increase [98]. Drought seems part of a systemic metabolic response when dryness significantly restricts CO_2_ availability inside leaf cells, raising the danger of secondary oxidative stress [17]. Root respiration and biomass may decrease during excessive soil drying, resulting in more significant drought-resistant wheat growth, physiological activity, and grain yield [21]. The drought-resistant wheat crop (*Triticum aestivum* L.) spring varieties should be favored over drought-sensitive wheat (*Triticum aestivum* L.) in dry settings [99,100]. The cell membrane stability (CMS) test can identify genotypes susceptible to drought stress. CMS and cell membrane integrity are indicators of resistance to limited water availability under water-stress situations. Lower CMS genotypes were more sensitive to water deficit stress and vice versa. Similarly, the CMS index is essential in breeding programs since it predicts drought tolerance or sensitivity requirements. Drought sensitivity is higher in genotypes with a low CMS value, but drought sensitivity is higher in genotypes with high CMs in wheat crops [23,101,102,103].

Even with limited water availability, CMS indicated a positive relationship between wheat crops (*Triticum aestivum* L.) tillering ability and grain output but a negative relationship between grain weight measured in kilograms (1000-grain weight) and grain yield [86]. As a side note, drought has been demonstrated to increase the oxidative process among plant species. This results in reduced membrane stability due to lipid peroxidation and, as a result, cell membrane damage [9].

## 4. Biochemical Responses under Drought Stress Conditions

Accumulating biochemicals such as proline, protein, sugar and glycine betaine (GB) improve crop production by scavenging ROS-generated oxidative stress [10]. Moreover, physiological processes including cellular respiration, rate of photosynthesis, mineral nutrition, enzymatic activities, and, Redox (oxidation/reduction) homeostasis are influenced by drought stress regimes. Likewise, biochemicals, including membrane lipo-proteins and DNA and cellular protein content, deteriorate under water-limited conditions [98]. Plants withstand drought stress regimes by developing various biochemical, structural, and molecular strategies, including the accumulation of certain osmolytes such as proline, proteins, sugars and glycine betaine. Applying salicylic acid improved drought-stress tolerance by upholding redox potential and activating proline biosynthesis [104,105]. Compatible solutes such as proteins, proline, glycine betaine, phenolic compounds, soluble sugars and organic acids accumulated chiefly in the cytoplasm in response to limited water availability by scavenging ROS, improving the water potential, and protecting biological molecules from lipid peroxidation [106]. Plant cells collect soluble chemicals during drought stress and increase cytoplasm viscosity. Under some situations, the content of these unique chemicals may become toxic, causing issues with enzyme development and the entire photosynthetic process [107]. The rate of regeneration of ribulose-1,5-bisphosphate, the maximum rate of ribulose-1,5-carboxylate, NADP-malic enzyme, phosphoenolpyruvate carboxylase, Rubisco, fructose-1,6-bisphosphatase, and orthophosphate-Di kinase pyruvate are all reduced as a result of the rapid decrease in “dry” photosynthesis [108]. Noncyclic electron transport is similarly lowered to satisfy the needs of decreased NADPH synthesis, ATP production, and ROS production. Different cultivars may respond and adapt differently to drought stress [109]. According to transcriptome studies, drought-tolerant and sensitive wheat genotypes may use distinct molecular processes to deal with drought stress. Differential expression of numerous drought-inducible genes involved in regulation, cell defense, and cellular component remodeling is one of the most noticeable changes [92]. According to transcriptome research, drought-tolerant and sensitive wheat (*Triticum aestivum* L.) genotypes may use molecular methods to cope with drought stress [69,84]. One of the most noticeable changes is the differential expression of several drought-inducible genes involved in cell defense regulation and cellular component remodeling [110]. While many of these genes are activated in drought-sensitive wheat (*Triticum aestivum* L.) genotypes and contribute to limiting drought impacts and perception, many of these genes are expressed constitutively in tolerant genotypes [111].

Furthermore, signal transduction and hormone-dependent regulation mechanisms change amongst *Triticum aestivum* L. genotypes [112]. Drought stress-tolerant genotypes perceive drought quickly and activate signal transduction pathways that trigger downstream components, helping them withstand drought stress [113]. When there is a lack of water, chemicals and metabolites including proline, glycine betaine (GB), and soluble sugar accumulate in the cytoplasm, assisting in osmotic adjustment and preparing the plant to cope with the adverse effects of oxidative stress in *Triticum aestivum* L. [62,114]. These metabolites are significant because their distinct biochemical processes promote plant tolerance—drought signaling results in crosstalk between various biological molecules and metabolites. Proline is an essential metabolite that accumulates in higher amounts in water-stressed environments [83].

### 4.1. Reactive Oxygen Species (ROS)

Water scarcity is the primary constraint on agricultural growth and development in irrigated and non-irrigated zones. This is because climatic conditions in irrigated and non-irrigated agricultural regions have changed [115]. ROS production is combined with a normal metabolic function in a drought-stressed climate, such as aerobic metabolism [116]. The reaction of plants to drought stress, whether through photosynthesis or other means, results in oxidative damage in proteins, lipids, and nucleic acids. Because plants are sessile creatures, they have devised techniques to assist them in surviving, adapting, or tolerating drought stress [58]. Under drought stress environments, increased ROS formation is unavoidable; phytotoxic levels of ROS are hazardous [117], resulting in cellular damage and even death [94,118]. However, they function as an essential signaling molecule at low concentrations, stimulating multiple stress-responsive pathways and initiating crosstalk between them. ROS-producing and scavenging enzymes and the antioxidant system fine-tune these for maintaining the cell’s redox state by removing or changing the intracellular ROS concentration [119].

### 4.2. Total Soluble Phenolic, Antioxidatant Enzymatic, and Osmolyte Regulation under Drought Stress Conditions

According to previous findings, there was a 100% increase in phenolic content under drought stress conditions [117,120,121]. Drought-stressed tomatoes had more total phenolic (46.4 mg GAE/100 g DM) than well-watered tomatoes [122]. Total phenolic rather than individual polyphenol concentrations were used in this study because of the wide range of phenolic compounds and the structural diversity of phenolic compounds [123]. Food polyphenol content cannot be determined using a single method, and the Folin-Ciocalteu reagent can be affected by other reducing agents, such as ascorbic acid [124]. High phenolic compounds in tomato fruits protect cells from oxidative damage. Peppers are a popular vegetable worldwide [14,125]. Drought stress reduces pepper fruit pithiness and reproductive development parameters; however, antioxidant activity was boosted after 45 days of blooming [79]. The coordination and management of multiple antioxidant enzymes in tea plants during drought stress is not well understood; despite all the stressful situations, foliar antioxidant content was noticed. Chemically reactive oxygen species are scavenged by enzymes that maintain membrane integrity and modify the osmotic pressure via signaling pathways that regulate gene expression and transcription [126].

Maize (*Zea mays* L.) crops under drought stress had the highest levels of antioxidant enzymes (POD), hydrogen peroxide (H_2_O_2_), glutathione (GSH), proline, and malondialdehyde of any crop tested (MDA) [86,127]. The finishing purpose of this study, according to the authors, was to assess the number of antioxidant chemicals discovered in the flesh of tomato fruits that had either been well-watered or had been subjected to a 10-day drought cycle throughout their development [128]. GPX produces lignin, guaiacol, and pyrogallol, which function as electron donors to scavenge hydrogen peroxide inside and outside the cell. Many studies have shown that GPX levels increase in drought-stricken plants like wheat crops (*Triticum aestivum* L.) [58]. The report concluded that drought stress increases GPX activity in rice and has been extensively researched and confirmed as a helpful screening approach for tolerance characteristics [129]. Proline is known for its vital role in osmoprotectants [130]. It is suggested that proline regulates cellular redox status and directly acts as a ROS scavenger under oxidative stress conditions. High proline concentration is associated with drought tolerance and a powerful defensive antioxidant system. The rainfed genotypes exhibited a greater proline concentration than irrigated or humid genotypes. Agricultural plants undergo various internal physiological processes [8,68].

Similarly, wheat (*Triticum aestivum* L.) cultivars with a high proline content in the leaves efficiently utilized water. Proline accumulates more significantly in response to various abiotic environmental challenges, including abiotic stress such as drought stress [131]. It is widely recognized that higher proline concentrations in agricultural plants cultivated under water-stress conditions relate to drought tolerance. Those drought-tolerant varieties have higher proline concentrations than drought-sensitive cultivars [132]. Many investigators identified a buildup of soil proline in the leaves of saline-stressed higher halophytic plants. However, plants subjected to drought stress showed significantly higher proline concentrations in the plants’ leaves, shoots, desiccating pollen, and root apical regions. Increasing the quantity of proline in the plant saves less water potential, resulting in the buildup of osmolytes in the osmoregulation process, allowing the plant to take up water for growth and metabolic activities [103,110,124].

The previous study explained that several antioxidant defence system enzymes’ activity changes when the wheat crop (*Triticum aestivum* L.) is exposed to oxidative stress caused by environmental stresses [133]. Guaiacol peroxidase, peroxiredoxins, SOD, CAT, GPX, ascorbate-glutathione cycle enzymes, including dehydro-ascorbate reductase, monodehydroascorbate reductase, APX, and glutathione reductase are among the enzymatic activities [15,134]. Tocopherols, carotenoids, and phenolic chemicals are non-enzymatic components, as are the primary cellular redox buffers ascorbate and glutathione. The wheat crop (*Triticum aestivum* L.), which is grown in the field and the lab, the activity of peroxidase, superoxide dismutase, ascorbate glutathione reductase, catalase, and guaiacol peroxidase, as well as the amount of ROS, were discovered [86,135,136]. Furthermore, multiple investigations show that abiotic stress has a genotype-specific effect on *Triticum aestivum* L., with different genotypes reacting differentially to the limited water supply. Drought-tolerant genotypes have a better antioxidant capability, which results in less oxidative damage [78,91]. Wheat crop (*Triticum aestivum* L.) responses vary by tissue type, duration, the severity of stress, and developmental stage, demonstrating the intricacy of ROS generation and detoxifying pathways and the impact of ROS on antioxidant systems [137].

## 5. Improvement of Drought Tolerance Using Molecular Tools

Rather than a qualitative feature, drought tolerance combines quantitative plant features regulated by several genes and other plant variables with minor individual impacts [138]. Understanding drought stress responses has necessitated the development of molecular regulatory understanding in recent years [139,140]. Transcriptome research, for example, has improved performance and aided the discovery of potential genes that might be used in plant breeding [141,142]. However, it was evident that the translational and post-translational machinery, particularly for immediate molecular activity during abiotic stress adaptation, is essential [112]. Understand stress-induced signal receipt and transduction, translational movement, and induced protein levels. In addition to transcriptome investigations, proteomics has emerged as the most direct and consequential approach for acquiring protein expression information on plants’ responses to drought stress [93]. Comparing proteomics of drought-tolerant and sensitive wheat (*Triticum aestivum* L.) genotypes is one technique for assessing the complexity of molecular pathways in wheat (*Triticum aestivum* L.) crop in response to drought stress [143]. In irrigation water shortage and climate change, efforts to enhance crop drought tolerance and related soil salinity are critical [144]. Specific chromosomal sites (quantitative trait loci (QTL) were connected to express traits using a combination of DNA fingerprints from various genotypes and phenotypic evaluations. Using marker-assisted selection (MAS) technology, some DNA markers have been linked to favorable QTLs [145]. Because of advancements in next-generation sequencing, the synthesis of many genetic markers, such as single nucleotide polymorphisms (SNPs) [146]; and insertion-deletions (InDels), provides a realistic option for increasing drought tolerance in cereal crops [147]. Drought-responsive genes and QTLs have recently been discovered in wheat (*Triticum aestivum* L.) crop, revealing that QTLs have been the focus of research over the last decade to identify the gene loci governing crops’ adaptive response to drought stress [148]. In addition to traditional and molecular plant breeding methods, the transfer of genes and gene regulatory sites vital for plant water management has emerged as an essential strategy [149]. Candidate genes have been thoroughly investigated in transgenic approaches [150].

In the previous research, many drought stress response genes were discovered and introduced into cultivated plants [132]; drought-resistant like *Triticum aestivum* L., *Oryza sativa* L., and *Zea mays* L. transgenic crops. Only a few drought-resistant grain cultivars developed through genetic transformation have been approved commercially [106,138,151]. The cspB gene, which encodes the cold shock protein B, was introduced into maize to give drought tolerance [129]. The cspB transgenic plant retains RNA stability and translation during drought stress, maintaining normal cellular function [152,153]. More profound knowledge of interactions between growth-promoting microbes and plants is another promising approach to the abiotic stress problem in many plants (PGPM) [132,154]. Plants can be protected against abiotic stress’s adverse effects, mainly drought and salinity stress. The biotechnology approach may be used to improve plant-microbe interactions. Plants inhabited by genetically changed soil bacteria that overproduce trehalose benefit from genetically modified PGPM [20,54,155].

## 6. Phytohormonal Modulation under Drought Stress

Phytohormones play an essential role in the development and growth of plants and their responses to environmental stress [156]. While not all plant cells respond to hormones simultaneously, those genetically programmed to do so at certain moments throughout the plant’s growth cycle. Plants need hormones at certain times and sites throughout their development and reproduction [157]. Hormones must disengage their effects when no longer required. Plants may also chemically break down hormones, leading to death [158]. Plant hormones oversee regulating the levels of other plant hormones [159]. Plant hormones are among the most significant biochemical influencing plant development and yield production in various environments, including drought stress [160,161]. Plant hormones are essential in developing and growing a plant when under water deficit stress [162]. Water stressed-induced responses in plant growth regulators such as salicylic acid, gibberellins, Cytokinin, and abscisic acid have been observed [149]. Besides stress responses, phytohormones also control internal and external stimuli and signal transduction pathways. Difficulty growing plants and low output are caused by different abiotic stresses, with drought stress most prevalent worldwide [163,164].

For this reason, the drought tolerance mechanism understanding in plants is essential for enhancing drought resistance in plants. According to [109] the growing body of research, phytohormones appear to be critical signaling molecules that modulate various wheat plant (*Triticum aestivum* L.) development processes and growth stages when plants are subjected to drought stress. The production of phytohormones regulates wheat plant (*Triticum aestivum* L.) growth in response to drought stress [106,165].

### 6.1. Salicylic Acid

Johann Buchner, a German scientist, first isolated SA from the bark of a Salix species (willow tree) in 1928 and named the glucoside of salicylic alcohol “silicon” [126]. SA is a phenolic molecule generated by secondary metabolism [166]; that plays a role in many biological processes, including CO_2_ assimilation, antioxidation, stomatal regulation, and photosynthesis [167,168]. Though SA’s role in abiotic and biotic stress has been thoroughly studied, evidence of its impact on drought stress is limited. Several studies, however, suggest that it may have a role in drought stress by modifying regulating drought-related genes through transcriptional regulation and stomatal aperture; depending on the amount of SA utilized, drought tolerance and sensitivity are affected [156,169,170].

Similarly, a higher SA treatment concentration reduces maize plants’ capacity to withstand drought. Water shortage increased endogenous SA levels significantly in *Phillyrea angustifolia* L. plants [171,172]. SA (500 M) applied externally to drought-stressed barley enhanced stomatal conductance and CO2 assimilation, leading to a dry matter increase [173]. According to [174], SA controls proline production and maintains the cellular redox state in the *Brassica rapa* L. plant. According to Castro et al., the light-induced stomatal opening was reduced in plants with high SA levels and the siz1 mutant (impaired function in SUMO E3 ligase, SIZ1), minimizing water loss and giving drought resistance [175].

Similarly, drought stress tolerance, increased SA buildup, and lower stomatal conductance was observed in cpr5 and acd6 mutants. Furthermore, many essential proteins were revealed for drought stress physiology and metabolism by priming the wheat seedlings with SA (0.5 mM) [133]. Proteins such as carbohydrate metabolism, photosynthesis, antistress proteins, and the signaling cascade are differentially expressed in primed seedlings, resulting in drought tolerance and improved growth [176,177,178]. SA applied exogenously has also been found to boost plant drought resilience. Plants overexpress CBP60g (a transcription regulator of SA biosynthesis) are more sensitive to ABA, accumulate more SA, and have a robust drought resistance phenotype [179].

Applying Salicylic acid (SA) to the leaves has induced plant stress tolerance. Several studies have found that Salicylic acid (SA) has beneficial effects on plants in terms of resistance to salinity, drought, and high temperatures [105,180,181]. The previous results suggested that Salicylic acid (SA) helps plants adapt to abiotic stresses [182]. Salicylic acid (SA) and exogenously applied substances develop dry period resilience and upgrade the submerged plants’ development and harvest [126,183]. Under drought-stressed conditions, salicylic acid (SA) application increased wheat crops (*Triticum aestivum* L.) catalase activity [184]. Salicylic acid (SA) and its derivatives in foliar and seed treatments improved drought tolerance in drought-stressed wheat crops (*Triticum aestivum* L.) [179]. Purslane (*Portulaca oleracea* L.) was utilized as a model plant in this study to see how foliar salicylic acid (SA) affected plant drought tolerance. According to the findings, Salicylic acid (SA) promoted purslane (*Portulaca oleracea* L.) growth by improving the pigments of photosynthetic apparatus and secondary metabolites production; suitable solutes and gas exchanges [185,186].

### 6.2. Cytokinin and Auxin

Another prominent phytohormone is cytokinin, which functions critically in the plant’s life cycle [81,187]. This low molecular weight plant hormone was initially found in maize (*Zea mays* L.) and is now recognized to serve many essential roles in plant growth and development [188]. Isoprenoid cytokinin contains an isoprenoid-derived side chain and aromatic cytokinin, which has an aromatic side chain at the N6 terminus [189]. The investigation of [190,191] revealed the existence of meta-tooling, a very active growth component that belongs to aromatic CKs, suggesting that aromatic cytokinin is far more significant than PGRs. The adenine moiety and the side chain are modified during CK metabolism. The central location of CK synthesis is the root tips, from which it is delivered to xylem sap by transpiration pull in an acropetal manner [192]. Cell division control, photosynthetic sink strength, unit stability, cell differentiation, delayed senescence, nutrient absorption, flower and seed germination and development, and prevention of lateral root initiation are just a few of the many functions of cytokinin in plant physiological processes [14,193,194]. The first phytohormone identified, Auxin, impacts some plant processes, including cell dedifferentiation and differentiation, root morphology or architecture, geotropism, root growth, floral organ development, and seed dormancy [195]. Recently, a tangible link between auxin content and plant drought stress response has been discovered. It has also been shown that auxin homeostasis regulates ABA production and drought stress responses [196]. TAA transforms tryptophan to IPA, which is then converted to IAA by YUCCA (YUC) flavin monooxygenase-like proteins in the auxin biosynthesis pathway (Arabidopsis) [197]. Drought-stressed rice (*Oryza sativa* L.) showed considerably decreased transcript abundance of IAA biosynthesis genes (YUCCAs) but dramatically increased transcription of IAA conjugating genes [198].

### 6.3. Gibberellins

Gibberellic acids (GAs) (tetracyclic diterpenoid carboxylic acid) can enhance plant growth and development in a different stage of the life cycle by boosting the cell division and elongation [199]. The most bioactive versions of the other GAs generated by plants are GA1 and Gas [200]. The GAs hormone is related to drought stress tolerance and is associated with seed germination, stem elongation, and reproductive development in the rice (*Oryza sativa* L.) plant [201]. Growth inhibitors imparted drought resistance to plants by lowering endogenous GA production, providing the first evidence of GA’s role in abiotic stress tolerance. In growth-retarded plants GA- and deficient mutants, GA treatment corrected dwarf growth and stress tolerance responses [202]. Plastid, endoplasmic reticulum (ER), and cytoplasm are involved in gas generation, with trans-geranylgeranyl diphosphate being the starting point in the chloroplast [203]. The overwhelming evidence implies that dioxygenases control GAs synthesis and that GA2ox genes in plants are primarily vulnerable to abiotic stress. Inhibition of plant growth and development by gibberellins (GAs), which are carboxylic acids that can regulate plant growth and development, has been observed. Gibberellins (GAs) affect leaf growth, seed germination, stem lengthening, flower development, and trichome formation [204,205]. Genetically altered (GA) hormones may interact with other hormones and impact several developmental processes [206]. These interactions may entail both negative and positive regulatory activities. Gibberellins (GAs) are a type of endogenous hormone found in plants that regulate the development of the plant’s vegetative and reproductive systems [207]. When controlling stem elongation, the effects of gibberellins (GAs) processes on cell growth and division are critical [208]. Compared to the shoot, Gibberellin insufficiency promotes the partitioning of reserves to the root [209]. Impaired GAs biosynthesis causes significant changes in primary metabolism, mainly due to drought stress [158,205]. Gibberellin deficiency enhances water content maintenance, improving drought stress tolerance [210]. Gibberellins (GAs) deficient symptoms look phenotypically like drought stress symptoms [211]. Under prolonged drought stress, plants show reduced height, leaf development, and flowering/fruit development [212].

Dwarfed plants with diminished stem elongation, leaf development, aberrant flowering, and fruit set occur from a decrease in endogenous GA concentration [213]. Water deficit stress lowers the rate of gene expression involved in GA biosynthesis, lowering the amount of bioactive GAs produced [214,215]. Under drought stress conditions, gibberellin content can be reduced, resulting in decreased internode elongation based on the degree of Gas reduction. Plants with less elongation may be more suited to situations where drought stress is standard. It inhibits stem cell elongation and growth [214]. Because GAs are critical regulators of cell elongation, the goal of the previous research was to see if the loss in development caused by drought is linked to changes in GA metabolism or signaling [106]. Drought stress, we postulated, influences plant development and stem elongation through its interaction with GAs metabolism, based on earlier research [216]. As a result, the main aim of this study was to find out how water-deficient stress affected stem elongation and Gas metabolism-related gene expression in tomato plants [217].

### 6.4. Abscise Acid

**Figure 6:**
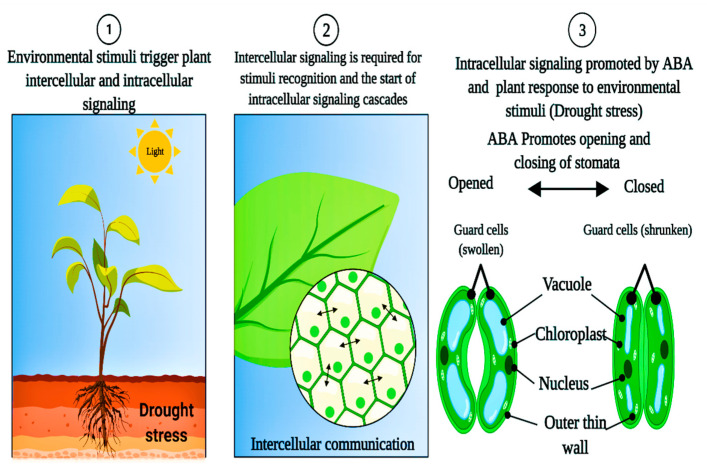
Abscisic acid (ABA) is vital for plant development and stress response. In response to biotic and abiotic stimuli, ABA transfer to guard cells triggers stomatal closure in leaves.

The natural plant stress hormone abscisic acid (ABA) regulates various physiological processes (Figure 6). Plants’ osmotic stress is linked to low water availability, triggering ABA production and adaptation mechanisms [156,218,219]. Abscisic acid production begins in the plastids once the plasma membrane receives stress signals, with the xanthorin transition to ABA being excluded; and happens in the cytoplasm. Most ABA is created in the roots and then transported to the plant’s upper portions via vascular tissues [220,221]. The former is a crucial player in expressing stress-responsive genes with the help of ABA under many situations, including osmotic stress [134,222,223]. Several receptors have been identified in the cytosol, plasma membrane, chloroplast envelope, and nucleus. Protein phosphatase 2C (PP2C) inhibits the action of non-fermenting sucrose 1-linked protein kinase 2 (SnRK2) proteins in plants with low ABA levels, resulting in dephosphorylation [224]. Antibiotics increase tolerance to drought in cotton (*Gossypium hirsutism* L.) plants by ABA, which regulates a stress-related gene [150]. In the Arabidopsis (*Arabidopsis thaliana* L.) plant, overexpression of the ABA-induced cotton gene (GhCBF3) leads to the high drought tolerance in transgenic lines by maintaining Ch, RW, and proline levels more significant than in the wild-type plant [225,226]. The stress hormone abscisic acid (ABA) is implicated in plants’ leaf abscission and abiotic stress [227]. ABA has the primary and critical role in plants’ developmental and physiological activities, including seed dormancy [228], tumor cell maintenance, stomatal opening, embryo morphogenesis, and fat and stored protein production. Abscisic acid affects the expression of protein-coding genes [229]. ABA is also required for root development and structural changes in nitrogen-deficient plants. Dehydrins, osmoprotectants, and protective proteins are all made by this enzyme. ABA plays two roles in drought stress: water balance and cell dehydration tolerance. Water balance is achieved in virtually all cells by controlling guard cells and the expression of genes that produce dehydration tolerance proteins [14,139,230].

Wheat crops (*Triticum aestivum* L.) with lower amounts of ABA in their leaves are more drought tolerant than those with higher proline levels [231]. When plants are drying out, soil moisture levels are more critical than leaf water levels, controlled mainly by ABA production in the roots [17,232]. Under drought stress, the phytohormone abscisic acid regulates crop morpho-physiology and biochemistry. Stomata closure is the most effective and essential response to ABA in drought-stressed crops [233]. Plants employ ABA as a signal molecule to help them cope with environmental stresses such as cold, salt, drought, heat, and phosphate deficiency in the olive tree (*Olea europaea* L.) [234]. Exogenous ABA treatment on leaves has been shown to elicit many adaptive changes in response to water scarcity, including the enhanced GR, SOD, APX, and CAT activity in tomato plants (*Solanum lycopersicum* L.) [81].

The exogenous ABA can also minimize ROS and increase cell membrane stability (CMS) to aid plants in their recovery after being subjected to stress [117,235]. Exogenous ABA spraying has been shown in some studies to improve plant stress tolerance in various crop species. However, research evaluating the responses of different *Zea mays* L. and *Glycine max* L. to drought stress using exogenous ABA and fluoridone is severely limited (ABA synthesis inhibitor) [236,237]. ABA substantially enhanced the activities of SOD and POD during drought stress, with a considerable drop after re-watering [235]. Under drought stress, ABA priming substantially raised the relative water content in both wheat cultivars [195]. Plant drought pathways use ABA as a primary stress sensor to improve the plant’s response to desiccation. The rise in ABA concentration coincided with the accumulation of lycopene and carotene in the fruits [238,239].

### 6.5. Ethylene

Gaseous phytohormone ethylene regulates the floral senescence, fruit ripening, petal and leaf abscission, and plant stress responses [240]. ET plays a vital role in biotic and abiotic stressors [28,241]. However, in these newly found activities of ethylene, there has been significantly less investigation on the drought stress response. According to a recent study, the dry shoot weight of six wheat genotypes ranging from tolerant to sensitive was more significant in the tolerant group under mild drought stress, related to an increase in ethylene [242,243]. Interestingly, several investigations on the influence of ethylene on stomata closure have shown contradictory results. For example, Arabidopsis eto1 mutants with higher ethylene accumulation have slower stomatal closure under drought stress conditions than control plants, even though ethylene has been considered to improve stomatal closure in guard cells [244,245]. More ethylene accumulates in the rice etol1 mutant, resulting in more drought-tolerant plants than OsETOL1 plants susceptible to drought stress treatment. Drought-tolerant transgenic plants were generated by modifying genes in the ethylene signaling pathway. Our findings underscore the need to understand and eventually use stress tolerance-related features in crops by interpreting ethylene signaling under abiotic stressors [246,247].

### 6.6. Jasmonates Acid (JAs)

Jasmonic acid (JA) is a phytohormone found in plants, and its active derivatives are known as jasmonates. It is essential in the fight against a variety of biotic and abiotic stressors [248]. Furthermore, JA is linked to improved root structure, pollen production, tendril coiling, and fruit ripening in many species [249]. Exogenously applied JA has improved plant performance and modulated stomatal dynamics in dry surroundings. JA signaling route and production have been extensively researched [250,251].

Nonetheless, in the absence of water, JAZ proteins are destroyed, resulting in active transcription factors such as MYC_2_, which up-regulate genes associated with stress tolerance [252]. Plant hormones, in most cases, do not function in a single route but rather interact with one another at different stages to control environmental and developmental pathways [253]. Signal transduction emerges in plants and may coordinate a complex set of events to adapt to a hostile environment. Jasmonates (JAs) are complex phytohormones created by the breakdown of lipids in the cell membrane in various plant species [162,254]. Plant growth regulators known as JAs may be found in almost every country. Jasmonates have also been shown to interact with other phytohormones to regulate plant growth and development and adapt to biotic and abiotic stimuli [250]. Seed dormancy and germination are affected by JAs in different ways. Jasmonates (JAs) treatment has negatively impaired seed germination in several species, including Solanum Lycopersicum, under water-stressed conditions. However, we know little about how JAs impact germination water deficit stress and salinity stress regimes [255,256].

## 7. Conclusions

Drought is a severe environmental stressor that threatens crop productivity worldwide. However, drought is more damaging during the reproductive and grain-filling stages (terminal drought). Terminal drought impacts grain set, pace, duration, yield production, and quality. Drought influences grain yield depending on the crop stage, length, and intensity. Drought-resistant genotypes and accompanying crop management practices can help reduce drought stress’s adverse effects. Improving drought resilience requires a thorough grasp of the impact of terminal drought. Although, research focusing on the physiological and molecular components of the drought response has helped improve wheat resistance to terminal drought. New advances in sequencing, marker creation, and genomic analysis have opened the door to tackling drought-resistant components. Drought stress has a long-term effect on CO_2_ absorption rates because it causes stomatal conductance to decrease. Deteriorated photosynthetic pigments, and restricted gaseous exchange, resulting in decreased plant growth and productivity. Plant growth, development, dry matter, and harvestable yield are all affected by drought stress, even though each species responds differently. Ramified root systems have been linked to drought resistance and high biomass production due to their capacity to collect more water from the soil and transport it to above-ground areas for photosynthesis. Many factors, including changes in photosynthetic pigments, influence the amount of water available to plants during drought stress regimes. A variety of roles in drought tolerance are played by carotenoids, one of two families of photosynthetic pigments. These roles include light-harvesting and oxidative damage avoidance, among other things. The phytohormone ABA influences drought stress responses and resistance in plants, which acts at the cellular and intercellular levels. However, it is unclear how plants detect drought stress and communicate that information into the cell to regulate ABA accumulation to withstand drought stress conditions.

## 8. Concluding Remarks and Outlook

Climate change and anthropogenic activity create a global danger to crop yield, exacerbated by shrinking agricultural areas, posing severe food security and safety challenge. Drought severely affects plant productivity and lowers the overall economic viability of agriculture. Many methodologies have been developed to challenge drought; each has its advantages and limitations. Though plants have an inherent defense system to deal with adverse environmental conditions, the genetic composition of the plant, the stage at which stress is identified, and the duration and degree of the stress all impact the plant’s reaction. The drought stress response is more than just a defense mechanism; it is also a means of achieving long-term development and ensuring a healthy ecological succession for future generations. Several studies have discovered several molecular markers associated with drought stress, with the phytohormonal syndicate having an important role. Because of their inter-crosstalk response, phytohormone signaling modules promote a complex cascade. The complicated reaction is enabled to improve the cellular potential to withstand adversities when multiple phytohormones are juxtaposed in a single frame of the event. As a result, proper drought stress response necessitates the interaction of these phytohormones and their communication and coordination. The discovery of PGRs crosstalk adds a new dimension to their previously well-understood functions and control. However, a thorough knowledge of these phytohormones’ molecular interactions remains completely unexplored. Although ABA helped relieve drought stress, the current work demonstrates the significance of hormone crosstalk throughout the drought stress response. Although most drought stress tolerance gene function research has been undertaken in the model plant Arabidopsis thaliana, the target gene(s) must be tweaked in economically relevant crops to benefit the end consumer directly.

## Data Availability

Not applicable.
